# The Molecular Twin artificial-intelligence platform integrates multi-omic data to predict outcomes for pancreatic adenocarcinoma patients

**DOI:** 10.1038/s43018-023-00697-7

**Published:** 2024-01-22

**Authors:** Arsen Osipov, Ognjen Nikolic, Arkadiusz Gertych, Sarah Parker, Andrew Hendifar, Pranav Singh, Darya Filippova, Grant Dagliyan, Cristina R. Ferrone, Lei Zheng, Jason H. Moore, Warren Tourtellotte, Jennifer E. Van Eyk, Dan Theodorescu

**Affiliations:** 1 Department of Medicine (Medical Oncology), Cedars-Sinai Medical Center, Los Angeles, CA USA; 2 Samuel Oschin Comprehensive Cancer Institute, Cedars-Sinai Medical Center, Los Angeles, CA USA; 3 Department of Oncology, Pancreatic Cancer Precision Medicine Center of Excellence, Johns Hopkins University, Baltimore, MD USA; 4 Betteromics, Redwood City, CA USA; 5 Department of Pathology and Laboratory Medicine, Cedars-Sinai Medical Center, Los Angeles, CA USA; 6 Department of Surgery, Cedars-Sinai Medical Center, Los Angeles, CA USA; 7 Department of Biomedical Sciences and Smidt Heart Institute, Cedars−Sinai Medical Center, Los Angeles, CA USA; 8 Department of Computational Biomedicine, Cedars−Sinai Medical Center, Los Angeles, CA USA; 9 Department of Urology, Cedars-Sinai Medical Center, Los Angeles, CA USA

**Keywords:** Machine learning, Pancreatic cancer, Predictive markers, Prognostic markers

## Abstract

Contemporary analyses focused on a limited number of clinical and molecular biomarkers have been unable to accurately predict clinical outcomes in pancreatic ductal adenocarcinoma. Here we describe a precision medicine platform known as the Molecular Twin consisting of advanced machine-learning models and use it to analyze a dataset of 6,363 clinical and multi-omic molecular features from patients with resected pancreatic ductal adenocarcinoma to accurately predict disease survival (DS). We show that a full multi-omic model predicts DS with the highest accuracy and that plasma protein is the top single-omic predictor of DS. A parsimonious model learning only 589 multi-omic features demonstrated similar predictive performance as the full multi-omic model. Our platform enables discovery of parsimonious biomarker panels and performance assessment of outcome prediction models learning from resource-intensive panels. This approach has considerable potential to impact clinical care and democratize precision cancer medicine worldwide.

## Abstract (web summary)

Theodorescu and colleagues describe a Molecular Twin approach that integrates multi-omic and computational pathology data from patients with pancreatic ductal adenocarcinoma using artificial intelligence to predict clinical outcomes.

## Main

Pancreatic ductal adenocarcinoma (PDAC) is one of the most aggressive malignancies. It accounts for 55,550 deaths in the United States and is expected to become the second-leading cause of cancer-related deaths nationally by 2030 (refs. ^1,2^). While only 30–40% of patients with PDAC present with localized disease and undergo potentially curative surgical resection after diagnosis or following neoadjuvant chemotherapy, most develop recurrence and succumb to their disease^3–5^. Despite advancements in molecular testing, serum carbohydrate antigen 19-9 (CA 19-9), first discovered in 1979, is presently the only US Food and Drug Administration (FDA)-approved biomarker widely employed for diagnostic management and preoperative prognostication of PDAC^6^; however, CA 19-9 has limitations, with a high false-positive rate due to other pathologic conditions and can result in false negatives in about 10% of the population^7^. Thus, there is an urgent need for improvement in new markers aimed at identifying patients most likely to be cured by surgery and/or respond to systemic therapies^8,9^.

One approach that could lead to such improvements is combining comprehensive molecular evaluation of the tumor and host with machine-learning (ML) models. Studies in other tumor types have employed ML and utilized various molecular analytes to predict therapy response and refine prognosis^10–12^. Most of these investigations, especially those on PDAC, have only focused on limited biological variables such as DNA and combined these with ML to determine whether findings can predict outcomes^13^. Multi-omic proteogenomic studies in PDAC have revealed unique phenotypes of PDAC, but they have shown limited ability to predict clinical outcomes^14^. Even if effective, the nature of such multi-omic analyses comes with high complexity and resource cost. Thus, an important consideration in the development of new predictive biomarkers is how to utilize the power of multi-omics to develop parsimonious panels of markers that would be both cost-effective and deployable in clinical practice in both resource rich and limited countries.

Here we use a multi-omic analytic platform that incorporates advanced molecular profiling beyond the examination of common analytes. Molecular profiling data were collected from both tumor and host samples and included computational pathology features. Multiple ML models were developed and applied to this dataset to test the hypothesis that this approach can provide biomarker panels that accurately predict DS after surgery in patients with resectable PDAC. Through recursive feature/analyte elimination, our approach was also able to provide a parsimonious model employing a limited number of features/analytes, which maintains a high degree of performance in prediction of DS compared to the full optimal models that we developed. Utilizing external samples/data from The Cancer Genome Atlas (TCGA), Johns Hopkins University (JHU) and Massachusetts General Hospital (MGH), we independently validated the power of our full and parsimonious ML models to predict DS. Through this analysis, we also discovered that among all analytes available in the preoperative setting, plasma protein is the most critical biomarker with substantial predictive power for survival and superior to CA 19-9. This work is a proof of concept for our Molecular Twin platform; a virtual, bioinformatic computational replica of the patient that can be updated and enriched in space and time with additional analyte types obtained longitudinally. While we utilize PDAC here, this approach is tumor-type agnostic, allowing it to potentially impact clinical care and scientific discovery across all cancers.

## Results

### Patient baseline demographics and specimen handling

**Fig. 1:**
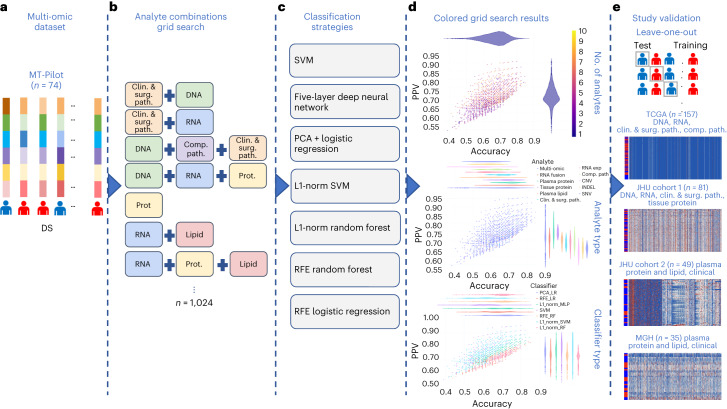
Study classification methodology overview. **a**, Combined multi-omic dataset of 6,363 processed features spanning clinical and surgical pathology, SNV, CNV, INDEL, RNA, fusion, tissue proteins, plasma proteins, lipids and computational pathology analytes. **b**, Construction of all possible analyte combinations (*n* = 1,024) via a drop-column importance approach to simulate availability of various combinations of analytes. **c**, For each analyte combination, seven independent ML models were trained for model evaluation, including SVM, principal-component analysis (PCA) + logistic regression, L1-normalized SVM, L1-normalized RF, five-hidden-layer deep neural network, RFE logistic regression and RFE RF. **d**, Input analyte combinations (*n* = 1,024) with seven modeling strategies per analyte combination produced 7,168 resulting grid search runs that were subsequently analyzed for predictive power, analyte composition and feature contributions for DS prediction. **e**, Each unique analyte combination and ML strategy was trained via leave-one-patient-out cross-validation approach. Single-omic and multi-omic models for DS prediction were validated using testing sets from four separate cohorts, TCGA, JHU cohort 1, JHU cohort 2 and the MGH cohort. Clin. & surg. path., clinical and surgical pathology; comp. path., computational pathology; prot., protein. Source data

Our Molecular Twin Pilot (MT-Pilot) cohort included 74 patients at clinical stage I (*n* = 47) and II (*n* = 27) with surgically resected PDAC between March 2015 and April 2019. Tumor specimens were collected at the time of surgery and plasma specimens were collected preoperatively. DS was recorded and treated as a binary end point at the time of analysis as of 21 October 2021. At this time, 45 (61%) patients were deceased. All demographic and clinical characteristics (Supplementary Table 1) were included as features for the clinical analyte in our multi-omic analysis. The surgical pathology information was obtained from a pancreas tumor resection. Tumor and plasma specimens were assessed for individual features by molecular profiling, including targeted next-generation DNA sequencing (NGS), full-transcriptome RNA sequencing, paired (tumor and normal from the same patient) tissue proteomics, unpaired (tumor from patients and normal unrelated controls) plasma proteomics, lipidomics and computational pathology. Analyte profiling yielded features that we used to validate single- and multi-omic models for predicting DS; a leave-one-out cross-validation approach was applied to the MT-Pilot cohort, whereas the four independent datasets: TCGA, JHU cohort 1, JHU cohort 2 and MGH were used to validate our ML models and feature panels developed based on the MT-Pilot data (Fig. 1).

### Clinical and surgical pathology features influence outcomes

**Extended Data Fig. 1:**
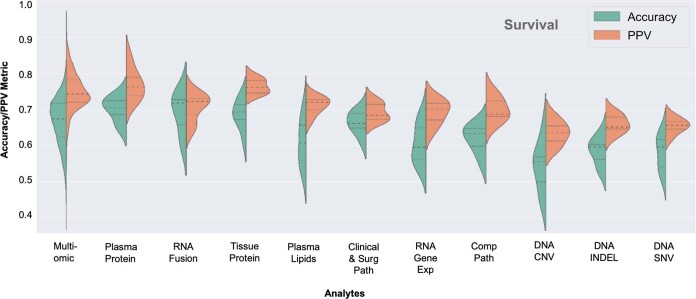
Top Single-omic and Multi-omic Performance for Disease Survival Prediction. Asymmetric violin plots showing accuracy and PPV distributions per analyte for predicting PDAC disease survival in decreasing order of accuracy (left to right) for multi-omic and single-omic analytes. PPV, Positive Predictive Value. Surg path, Surgical Pathology. Comp Path, Computational Pathology. Source data

The 331 clinical features, including surgical pathology features and chemotherapy treatment (Supplementary Table 1), as well as comorbidities (Supplementary Table 2) were analyzed using multiple ML models. When trained with these features, the random forest (RF) was the top performing model in determining DS and achieved an accuracy of 0.70 (95% confidence interval (CI) 0.60–0.81) and positive predictive value (PPV) of 0.71 (95% CI 0.60–0.82) (Table 1and Extended Data Fig. 1). The top features predicting outcome included comorbidities, such as hyperlipidemia, jaundice and pancreatitis, as well as surgical margin status (Supplementary Table 2), which are known in the PDAC field^15–17^. The model for DS was predominantly driven by comorbid conditions, which accounted for 306 of the 331 total features. The RF model was also trained using the remaining 25 features, which included known PDAC predictors such as previous chemotherapy and margin status. This model performed similarly to ones that included all clinical features (Supplementary Table 2). Notably, the top ten features of this model included surgical margin status, tumor grade and chemotherapy, which are known to influence patient outcome^18,19^.

**Table 1:** Top single-omic and multi-omic analytes for predicting disease survival in PDAC in the MT-Pilot cohort

Analytes	No. of samples	No. of features	TP	FP	TN	FN	ACC (95% CI)	PPV (95% CI)	Sensitivity	Specificity
Multi-omic	39	6,363	26	4	7	2	**0.85** (0.73–0.96)	**0.87** (0.75–0.99)	0.93	0.64
Plasma proteins	51	257	32	8	6	5	0.75 (0.63–0.86)	0.80 (0.68–0.92)	0.86	0.43
RNA fusions	57	29	35	12	8	2	0.75 (0.64–0.87)	0.74 (0.62–0.87)	0.95	0.40
Tissue proteins	49	1,130	32	10	4	3	0.73 (0.61–0.86)	0.76 (0.63–0.89)	0.91	0.29
Plasma lipids	51	406	34	12	2	3	0.71 (0.58–0.83)	0.74 (0.61–0.87)	0.92	0.14
Clinical and surgical pathology	74	331	47	19	5	3	0.70 (0.60–0.81)	0.71 (0.60–0.82)	0.94	0.21
RNA gene expressions	57	2,000	33	14	6	4	0.68 (0.56–0.80)	0.70 (0.57–0.83)	0.89	0.30
Computational pathology	71	819	34	11	13	13	0.66 (0.55–0.77)	0.76 (0.63–0.88)	0.72	0.54
DNA CNVs	72	648	43	20	4	5	0.65 (0.54–0.76)	0.68 (0.57–0.80)	0.90	0.17
DNA INDELs	72	126	39	17	7	9	0.64 (0.53–0.75)	0.70 (0.58–0.82)	0.81	0.29
DNA SNVs	72	611	45	23	1	3	0.64 (0.53–0.75)	0.66 (0.55–0.77)	0.94	0.04
CA 19-9 presurgery	63	1	17	15	20	11	0.59 (0.47–0.71)	0.53 (0.40–0.65)	0.61	0.57

Single and multi-omic analytes predicting DS are listed in decreasing order of predictive performance for DS, arranged by accuracy and PPV. For each analyte, the of number of samples available and features extracted for that respective analyte are shown. The predictive performance for each analyte is based on the best-performing model. TP, true positive; FP, false positive; TN, true negative; FN, false negative; ACC, accuracy. The bold ACC and PPV values indicates the best-performing analyte.

### DNA analysis reveals alterations with prognostic importance

Point mutations and insertion/deletion (INDEL) polymorphisms are common in established PDAC oncogenes and tumor suppressor genes^20^. Tissue samples were processed for 611 somatic single-nucleotide variants (SNVs), 648 copy-number variations (CNVs) and 126 INDELs. These features were then used in patient DS prediction models (Supplementary Table 3).

Using SNV features, the top-performing model to determine DS was RF, with accuracy of 0.64 (95% CI 0.53–0.75) and PPV of 0.66 (95% CI 0.55–0.77) (Table 1 and Extended Data Fig. 1). In models evaluating SNVs, we found that alterations in *RAD51*, *IL6R*, *FGF20* and *SOX2* genes were the top DS predictors (Supplementary Table 3) and their associated signaling pathways have important prognostic implications in PDAC^21–23^. In addition, we found genes, such as *RIT1*, that were top predictive DS markers identified by our model and not previously associated with PDAC prognosis or targetable pathways.

Using CNV features, the top-performing model to determine DS was an RF model with an accuracy of 0.65 (95% CI 0.57–0.80) and PPV of 0.68 (95% CI 0.57–0.80) (Table 1and Extended Data Fig. 1). The top CNV features for DS are noted in Supplementary Table 3. Notably, we found that *FOXQ1* and *KDM5D* were top predictors associated with DS. Both are markers for PDAC prognosis and potential therapeutic targets^24–26^. In our cohort, the four commonly mutated genes, *KRAS*, *TP53*, *CDKN2A* and *SMAD4* (ref. ^27^), were included among a total of 126 specific INDEL features and were learned by multiple ML model types. The top model predicting DS was RF with an accuracy of 0.64 (95% CI 0.53–0.75) and PPV of 0.70 (95% CI 0.58–0.82) (Table 1 and Extended Data Fig. 1). The top features in the model included mutations of *TP53, CDKN2A* and *SMAD4* (refs. ^28,29^), which have been shown to correlate with poor prognosis and more aggressive phenotypes of PDAC.

### RNA signatures of drug resistance impact prognosis

Whole-transcriptome sequencing was performed on 72 of the 74 formalin-fixed paraffin-embedded (FFPE) tumor tissue samples. To optimize the most predictive features, we first ran a differential expression analysis between cancer and noncancer samples from the GTex Consortium to select RNA gene transcripts for downstream modeling^30^. The top-performing model to determine DS was L1-normalized RF, which yielded an accuracy of 0.68 (95% CI 0.56–0.80) and PPV of 0.70 (95% CI 0.57–0.83) (Table 1 and Extended Data Fig. 1). In our top model for DS prediction the *NFE2L2* and *LRIG3* genes were the two top features (Supplementary Table 4). Recent investigations have shown that the NRF2 pathway through NFE2L2 regulates resistance to drugs and immunotherapy^31,32^. Additionally, a total of 29 RNA fusions were analyzed using multiple model types (Supplementary Table 4). The top performing model featuring RNA fusions to determine DS, was support vector machine (SVM) with an accuracy of 0.75 (95% CI 0.64–0.87) and PPV of 0.74 (95% CI 0.62–0.87) (Table 1 and Extended Data Fig. 1).

### Plasma proteins are critical biomarkers in survival prediction

Proteomics and lipidomics analysis initially generated 3,777 tumor tissue proteomic, 1,051 plasma proteomic and 939 lipidomic features (Supplementary Table 5).

Using tissue protein features, the top performing model to predict DS was RF model with accuracy of 0.73 (95% CI 0.61–0.86) and PPV of 0.76 (95% CI 0.63–0.89) (Table 1 and Extended Data Fig. 1). For plasma protein features, the top-performing model for DS was the five-hidden-layer-deep neural network model with an accuracy of 0.75 (95% CI 0.63–0.86) and PPV of 0.80 (95% CI 0.68–0.90) (Table 1 and Extended Data Fig. 1). Among DS predictive plasma proteins, we identified ANXA1, which is an important emerging player in pancreatic carcinogenesis and PDAC drug resistance^33,34^. The top performing model using plasma lipid features to determine DS was the RF model with an accuracy of 0.71 (95% CI 0.58–0.83) and PPV of 0.74 (95% CI 0.61–0.87) (Table 1 and Extended Data Fig. 1). The top plasma lipidomics features for DS were driven by diacylglycerols and cholesteryl esters (Supplementary Table 5).

As discussed above, CA 19-9 is routinely utilized in clinical practice at PDAC diagnosis, pre- and postoperatively to assess disease biology, treatment response and prognosis. CA 19-9 readouts obtained at diagnosis, before surgery and postoperatively, were learned by the RF model, but the DS prediction had a low accuracy (0.59–0.64, 95% CI 0.47–0.76) and PPV (0.52–0.61, 95% CI 0.40–73) across all time points (Supplementary Table 6).

### Predictive nuclear morphology via computational pathology

**Fig. 2:**
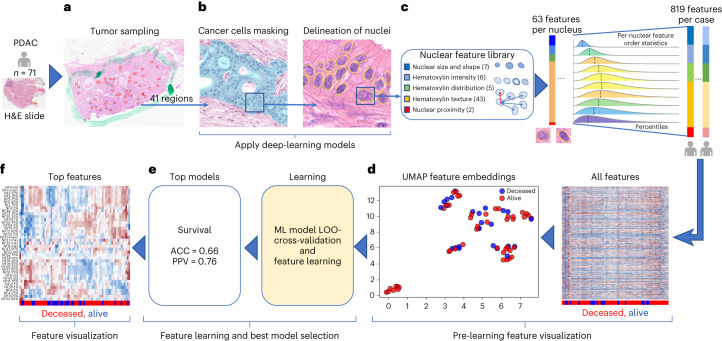
Computational pathology pipeline. **a**,**b**, Images of random tumor nests selected by a pathologist in digital H&E slides (**a**) are sent for processing by deep-learning models to provide a mask of tumor cell nuclei (**b**). **c**, Downstream nuclear feature extraction and formation of order statistics of morphology and H&E staining features in nuclei under the mask in patients from the cohort. **d**, Patient-level visualization of extracted features by the clustergram (right) and UMAP feature embeddings (left) plots. **e**, Feature learning by multiple ML models using a leave-one-out (LOO) cross-validation strategy to identify the models that can predict survival with the highest accuracy. **f**, Visualization of the top features learned by top survival prediction models. The top features were selected based on the feature importance learned by the models. Source data

The 71 hematoxylin and eosin (H&E)-stained PDAC tissue whole-slide images (WSIs) were evaluated by a artificial intelligence (AI)-based computational pathology pipeline (Fig. 2). The pipeline included two convolutional neural network models: a model to mask-out cancer cells (Fig. 2a) and a model to delineate nuclei (Fig. 2b). When tested on images from an independent set of 40 PDAC cases, the cancer-masking model achieved 0.90 global accuracy, 0.784 mean intersection over union (mIoU) and mean F1-scores of 0.83 and 0.77 in identifying nontumor and tumor tissue pixels, respectively. Next, the pipeline was run on 2,908 regions (~41 ± 11 regions per case) randomly selected from the 71 WSIs in our cohort and automatically isolated 345,038 tumor cell nuclei (~4,860 nuclei per case). Nuclear morphology and texture were quantitated by a panel of 63 characteristics. Distribution of characteristics in each case was further summarized by 13 order statistics, yielding 819 features per case (Fig. 2c and Supplementary Table 7). A uniform manifold approximation and projection (UMAP) plot revealed clusters of cases with similar outcome (Fig. 2d) suggesting that some of the features in the panel bear prognostic potential. Using the leave-one-out approach and the 819 features per case, we cross-validated seven classification models for DS prediction. An RF with an accuracy of 0.66 (95% CI 0.55–0.77) and PPV of 0.76 (95% CI 0.63–0.88) (Fig. 2e) performed the best. Throughout all validation steps, features learned by the top models were ranked based on the impact on predicting the outcome and the frequency of occurrence of impactful features measured. Impactful features that occurred in at least 10% of validation steps were considered top features. The 17 out of 39 top features to predict survival in Fig. 2f originated from the same 10 out of 63 nuclear characteristics in Fig. 2c.

**Extended Data Fig. 2:**
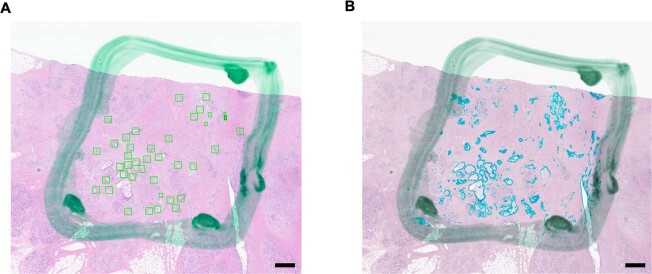
AI Modeling of Tumor and Stroma. **(a)** H&E slide with the tumor area and regions of interest (ROIs) marked by pathologist (WT). Frames of selected regions were box-outlined and saved in a database file to assure reproducibility of the experiment.; **b)** Same area with the cancer cells mask (cyan) predicted by our AI model. Segmentation of cancer mask by DeepLabV3Plus was performed 10 times. Each time the exact same mask was outputted. Scale bar is 1mm.

To assess whether the computational pathology-based prediction of DS could benefit from the inclusion of percent of stroma or cancer to stroma ratio in our samples, we applied our pipeline (Fig. 2b) to the cancer regions marked by our pathologist (W.T.) and measured the proportion of tumor pixels (pCA), stromal pixels (pST) and the ratio of these two (*r* = pCA/pST) in the regions (Extended Data Fig. 2a,b). No statistically significant difference in pCA (*t*-test *P* value = 0.3) and *r* (*t*-test *P* value = 0.257) was found when tumors associated with poor survival (DS = 1, *n* = 28) were compared to those with better survival (DS = 0, *n* = 43). As no difference was observed, we did not incorporate the above features into the computational pathology analyte. Regardless, we found that the percentage of stroma is significantly larger in tissue after neoadjuvant therapy, which can occur following neoadjuvant therapy. Additionally, the percentage of cancer was smaller in tissue after neoadjuvant therapy, which is the intent of neoadjuvant therapy (Supplementary Table 8).

### Multi-omic analysis suggests hierarchical complementarity

The 6,363 individual features from the single-omic sources were combined and analyzed using seven independent ML models cross-validated with a leave-one-patient-out technique (complete multi-omic feature dataset: Table 1 and Source Data Fig. 1). Each single-omic source and multi-omic combinations were evaluated using all ML models. The hyperparameters of each model were fixed at the initial design of the study to prevent over-optimization and overfitting due to the small cohort size. The top model for prediction of DS was the multi-omic model, which had an accuracy of 0.85 (95% CI 0.73–0.96) and PPV of 0.87 (95% CI 0.75–0.99), followed by single-omic analyte models that learned plasma protein, RNA fusions, tissue protein, plasma lipids, clinical and surgical pathology, RNA gene expression, computational pathology, DNA CNV, DNA INDELS and DNA SNV features in decreasing order of model prediction accuracy (Table 1 and Extended Data Fig. 1).

The accuracy and PPV performance yielded by single-omic models suggest that each single-omic analyte in isolation carries some predictive power and thus potential clinical utility. The best predictors of DS were plasma proteins leading to development of a model with an accuracy of 0.75 (95% CI 0.63–0.86) and PPV of 0.80 (95% CI 0.68–0.92). The model learning only presurgery CA 19-9 achieved an accuracy of 0.59 (95% CI 0.47–0.71) and PPV of 0.53 (95% CI 0.40–0.65) and it was considered the worst among all the single-omic models. As observed in the top two rows of the model performance (Table 1), the top multi-omic models outperformed the single-omic ones in accuracy (by 10–21%) and PPV (by 7–19%) in predicting DS, suggesting complementarity and information gain across analytes when combined under the multi-omic analytical approach. On the other hand, the multi-omic models had a larger dispersion of accuracy and PPV, when compared to the single-omic models (Table 1 and Extended Data Fig. 1) likely resulting from the involvement of a much larger set of features available for training.

**Fig. 3:**
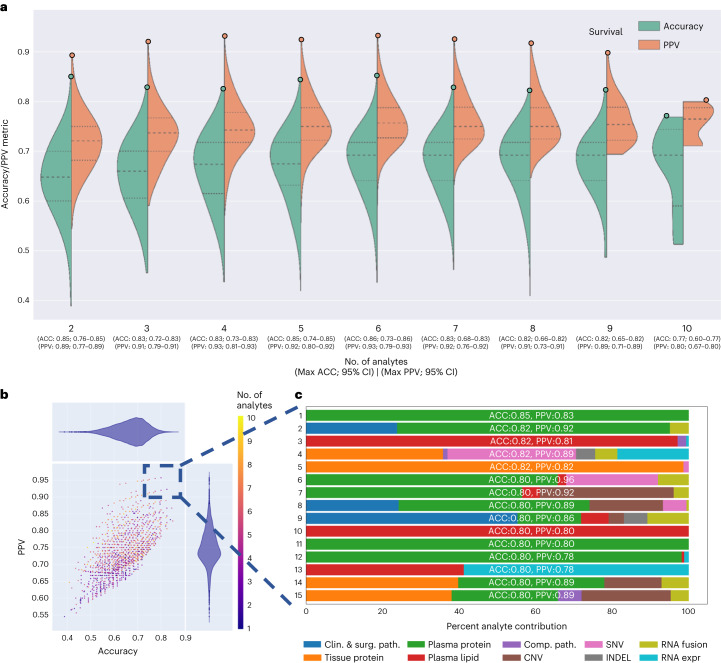
Multi-omic performance by number of analytes and contribution. **a**, Asymmetric violin plots showing ACC and PPV distributions for multi-omic survival models, segmented by number of analytes in the multi-omic combinations. Green and orange dots represent ACC and PPV of multi-omic analyte combinations with increasing number of analytes right to left on the *x* axis. **b**, Multi-omic grid search model results for DS; number of analytes 1–10 represent plasma protein, RNA fusions, tissue protein, lipids, clinical and surgical pathology, RNA gene expression, computational pathology, DNA CNV, DNA INDEL and DNA SNV. The *y* axis shows PPV and the *x* axis shows ACC. **c**, Top 15 multi-omic models for prediction of survival with percent contribution of each individual analyte listed in order of descending accuracy. Source data

The 1,024 individual analyte combinations (single and multiple) with all seven modeling strategies per analyte combination resulted in 7,168 grid search runs (Fig. 1). To establish per-analyte importance, the drop-column importance strategy was utilized and adapted, where each analyte’s set of features were dropped in their entirety. Using results from the 7,168 runs, we evaluated the model’s predictive performance, analyte composition and feature contributions (Fig. 3). Models trained with features from any 2–4 or 9–10 analytes were inferior in accuracy and PPV to the models trained with features from any 4–8 analytes (Fig. 3a).

Additionally, with the drop-column importance approach, we were also able to quantify the importance of each analyte category (Supplementary Table 9) and showed that the exclusion of any one analyte from the study generally reduced but did not substantially alter the performance; where the accuracy and PPV for DS prediction were in the range of 0.85–0.83 and 0.84–0.83, respectively.

Next, we focused on the top 15 multi-omic models for DS prediction (Fig. 3b), which were those with an accuracy >0.80 and PPV > 0.78. We plotted proportions of analyte’s features learned by each model (Fig. 3c) and observed that the top models had nearly similar accuracies and PPVs, however the proportions of contributing features varied across the top 15 models. The predominant feature contribution was from the plasma protein analyte (green bar, Fig. 3c).

### Multi-omic models provide biological insights into PDAC

**Fig. 4:**
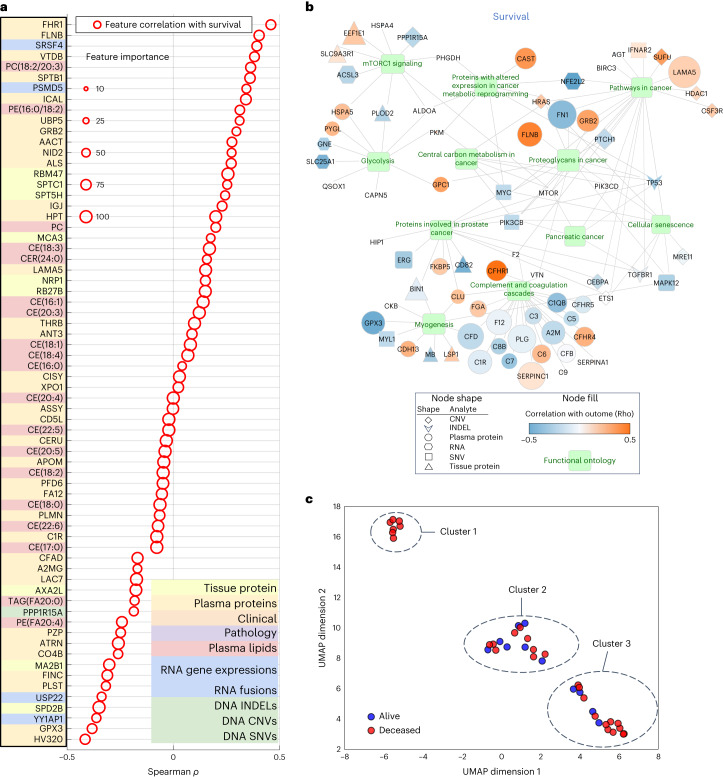
Biological relevance of top features in muti-omic model and clustering. **a**, Spearman correlation of top multi-omic features with DS. Size represents a feature’s relative importance to the top multi-omic model; the red color indicates whether the feature importance pertains to DS. **b**, Gene Ontology network visualization for most informative features from the multi-omic models. Selected functional pathways containing gene sets from multi-omic analytes are displayed as green nodes, with associated genes and measured analyte types represented by a specific shape (based on analyte) and colored according to the strength of a given analyte’s correlation to the outcome variable of DS. Size of a given analyte node is relative to the frequency with which that analyte was selected for the models, with larger analytes more consistently selected and no visible node indicating that the analyte was not selected as important for the DS outcome displayed. **c**, UMAP clusters of patients using molecular signatures consisting of all 6,363 multi-omic features, colored by survival. Source data

Given the relative paucity of predictive biomarkers and therapeutic advances in PDAC compared to other cancers, a notable exploratory objective of our study was to assess whether our platform can identify potential pathways and targets of therapy. Using a differentially expressed feature set, we were able to ascertain features to study objective Spearman correlations and the importance for all analyte features (Fig. 4a). By evaluating analyte contribution for each model, it was possible to generate ontology visualizations for protein, DNA and RNA as shown for the top multi-omic models for DS (Fig. 4b).

mTOR signaling, a known pathway in many tumors^34,35^ including PDAC, was found in the Gene Ontology network visualizations of the top multi-omic models^36^ (Fig. 4b). It has been targeted in PDAC alone and in combination with other agents^37^ with mixed results. Excluding mTOR, our Gene Ontology network visualizations revealed other clinically and biologically relevant pathways in PDAC, including glycolysis and cellular metabolism^38,39^.

**Extended Data Fig. 3:**
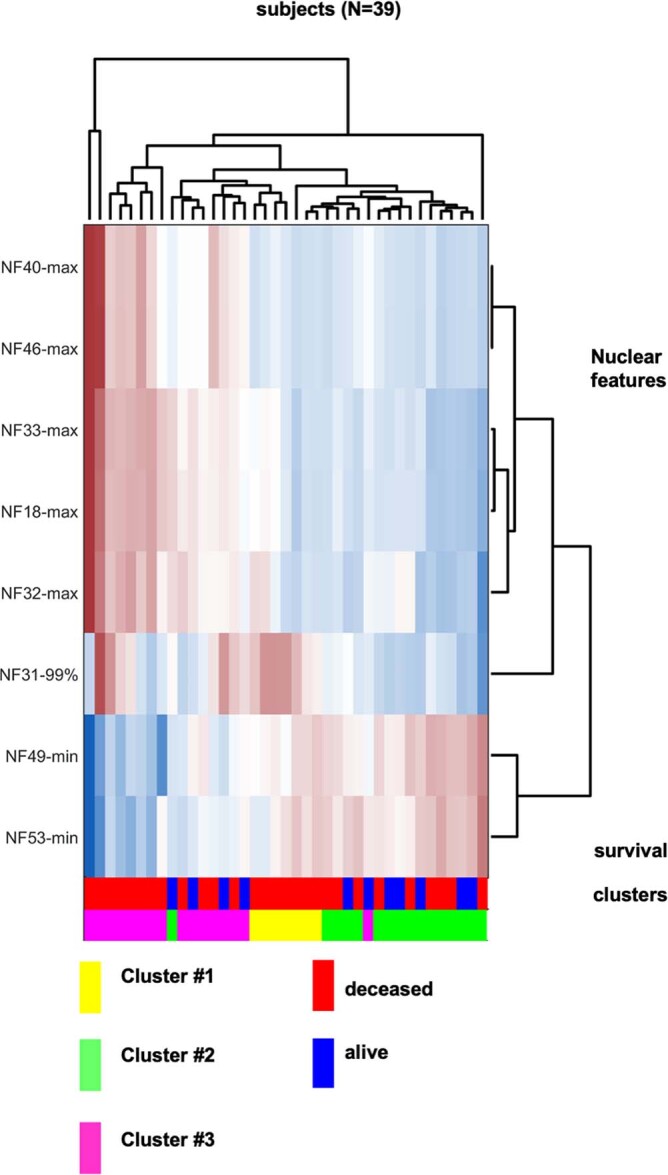
Hierarchical co-clustering of 8 features extracted from tumor cell nuclei. Cluster labels correspond to those in Fig. 4c. Cluster #1 represents patients homogeneous for their clinical outcome (all deceased) and multi-omic features. Cluster #2 represents a heterogeneous population with regards to clinical outcome while cluster #3 represents a more homogenous population compared to cluster #2. Notably, in cluster #3, patients noted to be alive at the time of analysis were strongly predicted to be deceased by the model. Source data

To examine the relationship of tumor to outcome heterogeneity, all 6,363 features across all analytes were used to create patient-level clustering based on multi-omic molecular signatures and labeled for DS (Fig. 4c). Cluster 1 represents patients homogeneous for their clinical outcome (all deceased). To better understand the association of the heterogenous clusters, (2 and 3), with other clinical and computational pathology features, we compared the expression of a feature in one cluster to that in the two other clusters combined using *t*-test or Fisher’s test. This analysis revealed proportions of relevant features (*P* < 0.05) in each analyte (Supplementary Table 10), where except for computational pathology, no other analyte contained features that were present in all three pair-wise comparisons. Subsequently, we used one-way analysis of variance, which identified eight differentially expressed computational pathology features (Supplementary Table 11). These eight features were then analyzed by the Tukey–Kramer test for multiple comparisons. No feature was significantly different between the three clusters, but there were features that differed between two clusters. Furthermore, hierarchical clustering of 39 subjects characterized by the eight computational pathology features (Extended Data Fig. 3) suggested that they strongly contributed to the formation of clusters 1, 2 and 3. Together, these findings suggest that with more patients and with prospective iterative analysis over time, our approach will result in progressively more accurate predictions especially for patients who fit membership in specific clusters (for example, cluster 1) and deeper insight into what features are critical to individual patient clusters.

### The parsimonious multi-omic models for disease survival

**Fig. 5:**
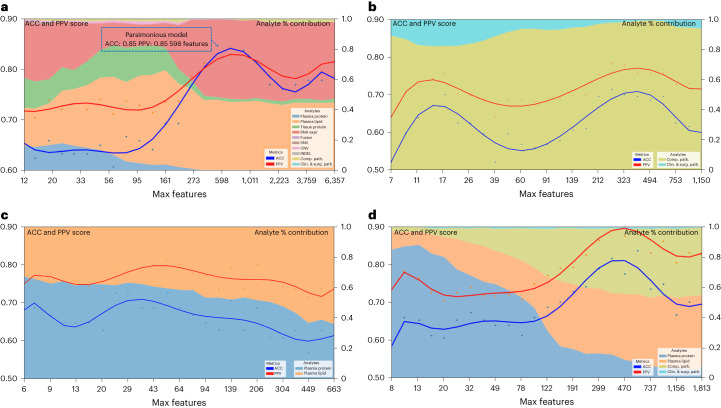
Performance of parsimonious multi-omic models and analyte contribution for disease survival. **a**, Parsimonious model of all multi-omic features and full dataset. The blue dotted line box indicates the parsimonious model at the inflection point. **b**, Clinical and surgical pathology and computational pathology analytes only. **c**, All plasma analytes (lipidomics and protein) only. **d**, All clinical and surgical pathology, computational pathology and plasma analytes (lipidomics and protein) only. Left *y* axis shows accuracy and PPV score: multi-omic model performance across feature reduction steps by restricting the maximum selectable features during model training. The *x* axis shows the number of maximum features at each reduction step. The right *y* axis shows the analyte percent (%) contribution: each analyte’s aggregated absolute feature weight contribution at each feature reduction step. Source data

The complementarity of analytes observed in multi-omic models in Table 1 and Fig. 3, suggested that a parsimonious multi-omic model offering similar predictive performance to models with larger and more complex analyte compositions could be developed. If true, the global public health and societal impact would be consequential as it would potentially begin the process of democratizing precision cancer medicine, especially to areas of the world with limited financial and technical healthcare resources. To test this hypothesis, we started with the complete multi-omic feature space of 6,363 features and trained an RF model for DS utilizing a recursive feature elimination (RFE) strategy such that at each step the least-informative features were eliminated from further model iterations (Fig. 5a). Most notably, Fig. 5a highlights the inflection point of the ‘parsimonious model’ location on the curve (accuracy of 0.85, PPV of 0.85) learning only 589 multi-omic features. Further, the contribution of respective analytes to the parsimonious model remains mostly stable across iterations after the inflection point, with plasma lipids and RNA being the most relevant; however, note that plasma (proteins or lipids) alone can provide accurate prediction with fewer features. This opens the possibility that a screening of plasma could eventually be used for decision-making regarding pancreatic surgery.

Trying to examine the potential of this approach for eventual globalization of precision oncology, we assessed specific limited analyte combinations and feature sets that could be applied to our parsimonious model. These analytes were selected based on criteria of standard availability (pathology specimens or clinical data including surgical pathology) or easily obtained (plasma lipids or proteins) as part of the diagnostic workup. Using this approach, we identified accurate parsimonious models that learned features from clinical, surgical pathology and computational pathology analytes (Fig. 5b), all plasma analytes (lipidomics and protein) (Fig. 5c) and clinical, combined with computational pathology and plasma analytes (Fig. 5d) and which had similar accuracy and PPV to the models that learned features from the entire set of 6,363 features in Fig. 5a.

### Validation of RNA markers as predictors of survival

**Extended Data Fig. 4:**
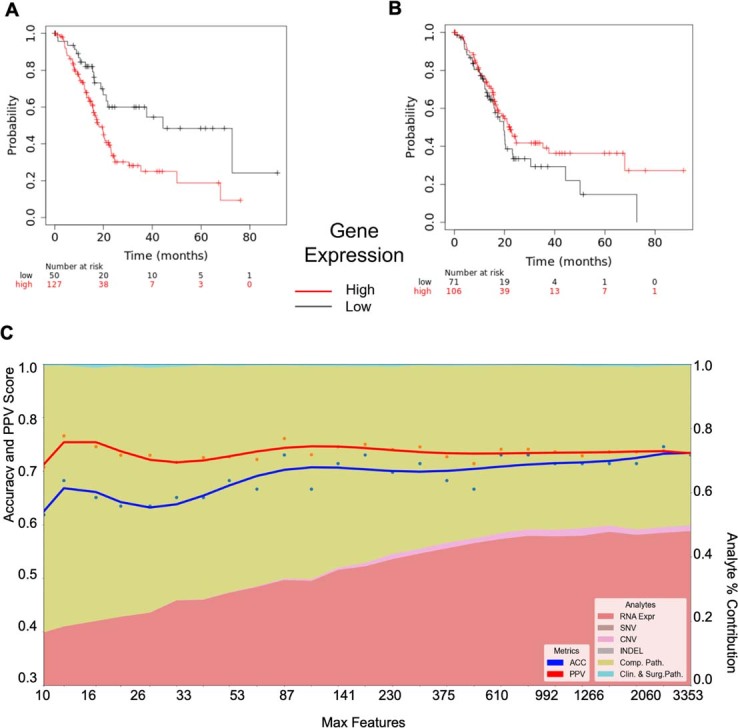
Validation of the Single-omic and Multi-omic and Parsimonious Models on TCGA. Validation of RNA gene signatures for disease survival: (**a**) 39 gene signature of poor survival (HR = 2.17, [1.28-3.66], logrank p = 0.0031) (**b**) 40 gene signature of improved survival (HR=0.74 [0.49-1.12], logrank p = 0.15) (**c**) Parsimonious model of clinical, DNA (CNV, INDEL, SNV), RNA gene expression and computational pathology in the original cohort used to select optimal 202 analytes (peak) for validation in TCGA. Multi-omic model performance across feature reduction steps by restricting the maximum selectable features during model training. Left y axis - Accuracy and PPV score: multi-omic model performance across feature reduction steps by restricting the maximum selectable features during model training. X axis – number of maximum features at each reduction step. Right y axis - Analyte Percent (%) Contribution: each analyte’s aggregated absolute feature weight contribution at each feature reduction step. Source data

Whole-transcriptome sequencing and analysis was performed on 57 samples from our pilot cohort (Supplementary Table 4). Employing L1-normalized RF modeling, RNA gene transcripts significantly (*P* ≤ 0.05) predicting survival (*n* = 79) were used to develop gene signatures for improved (positive Pearson and Spearman rho for survival, *n* = 40 genes) and for poor (negative Pearson and Spearman rho for survival, *n* = 39 genes) survival (Supplementary Table 12). These two signatures were evaluated in an independent dataset of 177 PDAC patients^40^ for their ability to stratify DS. High score of the signature composed of genes whose expression was associated with poor prognosis in our data (*n* = 39) was also associated with poor DS in this set (hazard ratio (HR) = 2.17, (1.28–3.66), log-rank *P* = 0.0031) (Extended Data Fig. 4a), whereas that of genes whose expression was defined as a good prognostic in our data (*n* = 40), had a trend toward improved DS (HR = 0.74 (0.49–1.12), log-rank *P* = 0.15) (Extended Data Fig. 4b). We also performed gene set enrichment analysis on the RNA transcripts used in the two signatures above (*n* = 79). Enrichr^41^ found numerous significant pathways (Supplementary Table 13) implicated in PDAC resistance and treatment-targeting, including interferon signaling, AMP-activated protein kinase (AMPK) and CXCR4 signaling pathways^42–45^. Together, these data independently validate the clinical relevance of our RNA expression discoveries.

### Validation of multi-omic models as predictors of survival

To further validate our single-omic, multi-omic and parsimonious analytes for DS prediction, we evaluated their predictive performance on the TCGA dataset, containing 157 evaluable samples that had at least one analyte type (Supplementary Table 1)^46^. As TCGA has data only on DNA, RNA, digital H&E slides and clinical analytes, our modeling had a reduced set of 3,423 total features compared to the 6,363 in our MT-Pilot cohort (Table 1 and Fig. 1e). The full 3,423 analyte model had an accuracy and PPV of 0.94 (95 CI 0.83–1.00) and 0.95 (95% CI 0.84–1.00) (Table 2 and Supplementary Table 14) for DS prediction with computational pathology, DNA SNVs and RNA gene expressions performing strongly in single-omic validation of DS (Table 2 and Supplementary Table 14).

**Table 2:** Top single-omic and multi-omic performance for predicting disease survival in PDAC: study validation cohorts

Analytes	No. of training samples	No. of validation samples	No. of features	ACC (95% CI)	PPV (95% CI)	Sensitivity	Specificity
**TCGA**							
Clinical and surgical pathology, DNA (SNVs, INDELs and CNVs) and RNA gene expressions,	45	109	3,024	**0.96** (0.88–1.00)	**0.98** (0.90–1.00)	0.95	0.98
Clinical and surgical pathology, DNA (SNVs, INDELs and CNVs), RNA gene expressions and computational pathology	45	33	3,423	0.94 (0.83–1.00)	0.95 (0.84-1.00)	0.95	0.92
DNA SNVs	72	126	351	0.94 (0.85-1.00)	0.96 (0.86-1.00)	0.95	0.94
Computational pathology	71	33	819	0.79 (0.68–0.89)	0.89 (0.78–0.99)	0.76	0.83
RNA gene expressions	57	152	1,974	0.76 (0.67–0.85)	0.80 (0.71–0.89)	0.76	0.76
DNA INDELs	56	120	43	0.72 (0.60–0.84)	0.82 (0.70–0.94)	0.68	0.77
Clinical	74	157	15	0.66 (0.57–0.75)	0.71 (0.63–0.80)	0.68	0.63
DNA CNVs	72	156	645	0.47 (0.40–0.54)	0.56 (0.49–0.63)	0.42	0.55
**JHU cohort 1**							
Clinical and surgical pathology, DNA (INDELs, CNVs and SNVs), RNA gene expressions and tissue proteins	39	81	3,270	**0.89** (0.83–0.95)	**0.91** (0.85–0.98)	0.84	0.93
Clinical and surgical pathology, RNA gene expressions and tissue proteins	40	81	2,480	0.75 (0.69–0.82)	0.72 (0.66–0.79)	0.76	0.74
RNA gene expressions and tissue proteins	46	81	2,466	0.72 (0.66–0.79)	0.69 (0.63–0.76)	0.71	0.72
RNA gene expressions	57	81	1,963	0.68 (0.62–0.75)	0.67 (0.61–0.74)	0.63	0.72
Clinical and surgical pathology, DNA (INDELs, CNVs and SNVs) and tissue proteins	45	81	1,307	0.65 (0.59–0.72)	0.63 (0.57–0.70)	0.63	0.67
Clinical and surgical pathology, DNA (INDELs, CNVs and SNVs) and RNA gene expressions	45	81	2,767	0.60 (0.54–0.67)	0.57 (0.51–0.64)	0.63	0.58
Tissue proteins	49	81	503	0.56 (0.50–0.63)	0.53 (0.47–0.60)	0.53	0.58
DNA (INDELs, CNVs and SNVs)	56	81	790	0.51 (0.45–0.58)	0.47 (0.41–0.54)	0.45	0.56
Clinical	74	81	14	0.38 (0.32–0.45)	0.35 (0.29–0.42)	0.37	0.4
**JHU cohort 2**							
Clinical and plasma proteins	41	47	255	**0.98** (0.83–1.00)	**0.92** (0.79–1.00)	1.00	0.97
Plasma proteins	51	47	251	0.98 (0.83–1.00)	0.92 (0.79–1.00)	1.00	0.97
Clinical, plasma proteins and plasma lipids	51	47	619	0.79 (0.63–0.94)	0.57 (0.44–0.69)	0.67	0.83
CA 19-9 presurgery	63	48	1	0.69 (0.57–0.81)	0.17 (0.04–0.40)	0.08	0.86
Plasma proteins and plasma lipids	51	47	615	0.55 (0.41–0.69)	0.30 (0.16–0.44)	0.58	0.54
Clinical	74	49	5	0.43 (0.29–0.57)	0.14 (0.02–0.26)	0.25	0.49
Clinical and plasma lipids	51	47	369	0.32 (0.20–0.44)	0.12 (0.00–0.25)	0.25	0.34
Plasma lipids	51	47	365	0.23 (0.12–0.35)	0.15 (0.03–0.27)	0.42	0.17
**MGH cohort**							
Clinical and plasma proteins	51	35	259	**0.91** (0.77–1.00)	**0.84** (0.71–0.97)	1.00	0.84
Plasma proteins	51	35	250	0.89 (0.76–1.00)	0.80 (0.69–0.91)	1.00	0.79
Plasma proteins and plasma lipids	51	35	614	0.74 (0.61–0.87)	0.68 (0.54–0.82)	0.81	0.68
CA 19-9 presurgery	63	32	1	0.62 (0.51–0.73)	0.60 (0.52–0.68)	0.60	0.65
Clinical, plasma proteins and plasma lipids	51	35	623	0.51 (0.41–0.62)	0.47 (0.33–0.61)	0.44	0.58
Plasma lipids	51	35	365	0.49 (0.36–0.62)	0.46 (0.30–0.62)	0.69	0.32
Clinical	74	35	10	0.40 (0.29–0.51)	0.37 (0.26–0.48)	0.44	0.37
Clinical and plasma lipids	51	35	374	0.37 (0.22–0.52)	0.35 (0.20–0.49)	0.44	0.32

Detailed results table for top survival models for each validation test cohorts TCGA, JHU cohort 1, JHU cohort 2 and MGH cohort. Single and multi-omic analytes predicting DS are listed in decreasing order of predictive performance for DS, arranged by ACC and PPV. For each analyte, the of number of samples available, trained and features extracted for that respective analyte are shown. The predictive performance for each analyte is based on the best-performing model. Analytes within each validation set are listed in decreasing order of survival accuracy. Bold ACC and PPV values indicate the best-performing analyte within each cohort.

Next, we examined the validity of our multi-omic parsimonious model on the TCGA dataset. Because this cohort had an overall reduced analyte set, we used an RFE strategy to retrain a RF model for DS on our cohort (MT-Pilot) and determined that the optimal (top of peak) parsimonious model employed 202 features out of 3,423 and had accuracy and PPV of 0.74 (0.63–0.85) and 0.77 (0.65–0.89), respectively (Extended Data Fig. 4c). Notably, when the model was applied to these same 202 features (Supplementary Table 15) in the TCGA dataset, it yielded an accuracy of 0.88 and PPV of 0.95 for DS prediction. Furthermore, in both our MT-Pilot cohort and the TCGA cohort, computational pathology and RNA gene expression were found to be primary analytes learned by the DS predicting models, with CNV and the clinical analyte providing minor additional improvement (Extended Data Fig. 4c). The signal dominance of RNA is not driven by expression of any single gene, but by a specific set of genes.

As TCGA lacked tissue proteomic level data, we sought an external dataset with tissue protein data, along with other critical single-omic informative analytes such as DNA, RNA and clinical data. We found an independent publicly available dataset^14^ named JHU cohort 1 that met these criteria. With DNA, RNA, clinical data and tissue protein analytes from our MT-Pilot cohort serving as the training set, we trained an L1-normalized RF model and applied it to this validation test set. This model predicted DS with an accuracy and PPV of 0.89 (95% CI 0.83–0.95) and 0.91 (95% CI 0.85–0.98), respectively (Table 2 and Supplementary Table 14). While a model trained on the tissue protein as a single-omic analyte had an accuracy and PPV of 0.56 (95% CI 0.50–0.63) and 0.53 (95% CI 0.47–0.60) in the JHU cohort 1 (Table 2 and Supplementary Table 14), addition of DNA, RNA and clinical analytes improved the predictive performance of the model and validated the multi-omics approach.

### Validation of plasma proteins as a preoperative biomarker

Through our multi-omic and parsimonious modeling of the MT-Pilot cohort, we discovered that plasma protein is an analyte that provides not only accurate prediction of DS in PDAC, but does so with the fewest features compared to other analytes. As a result of these findings, as well as the poor performance of CA 19-9 as a preoperative marker for decision-making regarding the benefit of surgery, we next sought to validate our findings solely on analytes that would be available to the clinical practitioner before surgery.

Besides the TCGA and JHU cohort 1, we utilized two more cohorts; JHU cohort 2 and the MGH cohort (Supplementary Table 1). They included similar stage I/II resected PDAC with clinical and demographic data collected longitudinally and preoperative plasma samples, including CA 19-9 obtained and analyzed as described above. Application of the L1-normalized RF model trained on the MT-Pilot data on the two cohorts showed that plasma proteins remained highly predictive of DS in both validation cohorts, with accuracy and PPV of 0.98 (95% CI 0.83–1.00) 0.92 (95% CI 0.79–1.00), respectively in JHU cohort 2 and 0.89 (95% CI 0.76–1.00) 0.80 (95% CI 0.69–0.91), respectively in the MGH cohort (Table 2 and Supplementary Table 14). The addition of clinical data to plasma protein improves the multi-omics model for DS prediction. Overall, preoperative plasma protein was highly predictive of DS among three separate independent datasets and provided a unique preoperative biomarker with significantly better predictive performance than routinely utilized CA 19-9 (Table 2 and Supplementary Table 14).

## Discussion

**Fig. 6:**
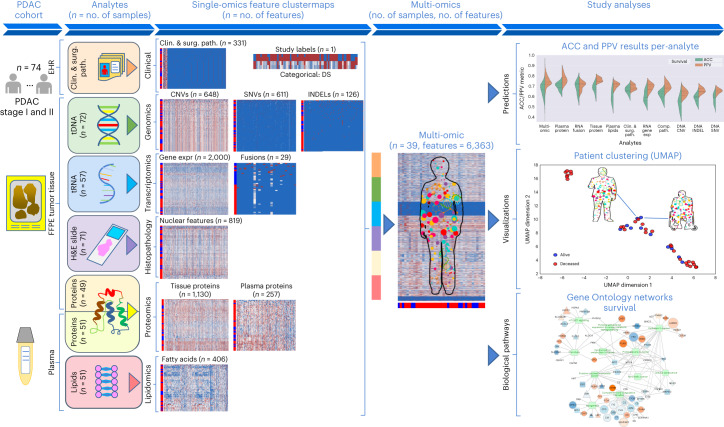
The Molecular Twin platform. The Molecular Twin platform ‘proof of principle’ applied to PDAC. Plasma and tissue samples from 74 patients with stage I/II resectable PDAC were subjected to targeted NGS DNA and whole-transcriptome RNA sequencing, tissue proteomics, plasma proteomics, plasma lipidomics and computational pathology to produce individual omics analytes. The 6,363 features were combined and served as input for seven different types of ML models to generate multi-omic biomarker models to predict clinical outcomes, provide patient-level clustering data and insight into possible therapeutic targets. EHR, electronic health record.

Here we demonstrate a ‘proof-of-principle’ Molecular Twin platform that incorporates multiple molecular, histopathological and clinical features from both host and tumor and a comprehensive machine learning-based multi-omic analysis to provide accurate clinical outcome prediction (Fig. 6). The Molecular Twin platform has allowed us to develop predictive multi-omic models and led to the discovery that plasma proteins are a highly predictive analyte for DS prediction in PDAC. Most notably, validation of the approach on four independent datasets have confirmed its value in predicting DS and revealed its superiority to CA 19-9. This approach has potential to substantially impact how we develop biomarkers in the future and in the case of preoperative markers, may have provided enough rationale to initiate clinical development and large-scale testing to determine its value in treatment decision-making. Finally, this platform, by virtue of its ability to generate parsimonious models has laid a foundation for the future democratization of precision oncology and thus reduce national and global disparities in its use.

Our study reveals that the multi-omic analytes incorporating individual single-omic sources are the most accurate clinical predictors of DS. We also show that multi-omic models with limited, but highly predictive analytes, perform just as well as the top multi-omic models with higher number of individual single-omic analytes. None of the top multi-omic models consisted of all ten available analytes. This reinforces the concept of complementarity and highlights the overlap of signal across analytes, suggesting that it may not be necessary to carry out the comprehensive ten-analyte workup to obtain accurate predictions. A strength of this platform is its resilience, allowing interchangeability and complementarity among analytes. This observation also suggests flexibility in analyte selection to approximate optimal predictive performance, with patient burden, efficiency, ease of testing, time and cost of analyte acquisition being other notable considerations. Many analytic techniques, especially comprehensive genomics, can be expensive as well as time and labor intensive; however, our study reveals single-omic sources employed in this platform, such as computational pathology-based features or plasma proteins which offer the opportunity to circumvent these challenges using near-term practical solutions with clinical implications in resource-poor geographies. In computational pathology analysis, features of nuclear architecture can predict survival^47–49^ in many cancer types, and our results were consistent with these reports. To extract features, computational pathology uses only H&E slides prepared to obtain routine pathology reports. As no special tissue processing or chemical reagents are necessary, the cost of measuring a feature through this platform is low. In addition, digital H&E slides can be sent for analysis through the cloud and results sent back to the requester as a multi-omic score generated by combining all other information on the patient electronically.

Studies employing smaller cohorts, for example one study with 14 patients, has shown that certain predefined plasma proteins can predict early recurrence^50^. Our study is more comprehensive, includes 74 patients and identifies more plasma proteins as critical predictors of DS. Plasma proteins within multi-omic panels also represent a unique opportunity for efficient, informative and clinically impactful testing as this specific analyte can be obtained quickly and preoperatively in a non-invasive manner. Although preoperative antigen testing, such as CA 19-9, continues to be routinely utilized in predicting tumor biology and survival^51^, we demonstrated that plasma proteins alone, and even more so when combined with other preoperative analytes such as clinical data, is superior to CA 19-9 alone. These results are not surprising as it is well appreciated that preoperative CA 19-9 has limitations that may contribute to its poor performance as a tool predicting DS^6^. Unlike CA 19-9, plasma proteins have the potential to inform subsequent therapeutic decisions involving surgery and chemotherapy. Our approach provided insight into molecular drivers and clinically useful markers of survival in PDAC; the latter findings helping to validate the value of our approach.

Multi-omic analysis across tumor types has been undertaken before, but not to this extent. One study employed a smaller number of analytes^13^, identified hurdles in multi-omic analyses and highlighted the high cost of genomic signatures in clinical practice. In our study, we sought to address the cost of data generation and technical sophistication, which are two of the major issues impeding the global use of precision therapy in cancer care. As a solution, we employed an RFE strategy to identify optimal features across analytes and developed the parsimonious model, which achieved an accuracy and PPV of 0.85 with 589 features, which was similar to the full multi-omic model trained with 6,363 features. We also found that plasma analytes were the dominant feature type of the parsimonious panel. The parsimonious model uncovered highly informative features, while simultaneously minimizing the number of required analytes without compromising performance.

A strength of our study is that we validated our findings in independent datasets of PDAC, including the TCGA cohort, two separate cohorts from JHU and a cohort from MGH. In our validation approach, we recognize that no single multi-omic model contains all ten single-omic analytes concurrently. This is an inherent shortcoming of our validation datasets as well as many currently available datasets, where none contain complete data of all ten single-omic sources that our original MT-Pilot cohort provided. Regardless, we externally validated our multi-omic panels with available and complete data where it was possible. Of note, for the potential democratization aspects of this work, the 202 highly predictive features learned from the MT-Pilot cohort data by the parsimonious model were sufficient to accurately predict DS in the TCGA cohort. Additionally, models learning single- and multi-omic panels incorporating plasma proteins were validated in two separate prospective test cohorts. This further supports the development of plasma proteins as a potentially clinically usable assay in PDAC.

Despite evaluating a larger number of patients and use of more comprehensive analyte panels, our study has several limitations. First, specific features of certain analytes have outcome relationships that are contradictory to published literature, likely a reflection of the sample size of our MT-Pilot cohort. Second, despite comprehensive panels, it still does not incorporate all accessible analyte sources. These include radiomic features extracted from preoperative computed tomography scans and microbiome features as just two examples; however, our platform is flexible so integration of additional sources of data is facile and has already begun. We also recognize that our molecular profiling of tissue was conducted on bulk tissue, which provides limited insight into tumor heterogeneity, but single-cell analysis may address this issue. Similarly, dissection of the tumor microenvironment with techniques such as multiplex immunohistochemistry (IHC) can provide further understanding into disease biology and improve the performance of our models. We also recognize the single time point multi-omic analysis as a potential shortcoming, and hence future iterations will incorporate longitudinal analyses to enhance the predictive potential of the platform, as well as discover additional markers for minimal residual disease or treatment response prediction. We are exploring these issues in upcoming iterations of the Molecular Twin.

This proof-of-concept, yet externally validated study, examined an aggressive malignancy, PDAC, that lacks robust predictive and prognostic biomarkers. The Molecular Twin represents the way forward for the discovery of promising predictive and clinically meaningful biomarkers, targets for treatment and ultimately tools to democratize and reduce national and global health disparities in the use of precision medicine across all of types of cancer.

## Methods

### Sample collection, processing and classification

Specimens were obtained from consented patients enrolled in the ethically approved study by Cedars-Sinai Institutional Review Board STUDY00000806 MT-Pilot study ‘Feasibility of extensive molecular profiling of pancreatic tumors: lessons for Molecular Twin’ and stored onsite in the Cedars-Sinai Medical Center Biorepository. Tissues were procured from surgical specimens as part of the standard of care. Blood samples were collected with routine blood work. The dates in which these samples were collected ranged from April 2015 to May 2019. Follow-up data were completed based on the standard of care. All cases were pancreatic cancer with the diagnosis of ductal adenocarcinoma. This was chosen based on the availability of FFPE and frozen tissue, buffy coat and plasma. FFPE and frozen tissues were collected following tumor resection and stored in the biorepository for future research use.

The Cedars-Sinai Medical Center Biobank and Pathology Shared Resource reviewed in-house cases and histologically confirmed PDAC from an initially assembled list. Specifically, fresh frozen tissue (tumor and adjacent normal) and FFPE tissues (tumor and adjacent normal) were identified. The biobank prepared each sample for genomic analysis (ten unstained slides per sample and one H&E). These slides were de-identified and sent to Tempus via overnight shipping for genomic and transcriptomic analyses as well as H&E slide digitization.

The following set of samples were sent to Tempus:

- 93 FFPE tumor samples (ten unstained slides and one H&E)
- 93 FFPE normal samples (ten unstained slides and one H&E)
- 93 blood samples (buffy coat at 500-μl aliquots)

### Clinical data variables for the cohort

Cedars-Sinai Medical Center Proteomics and Metabolomic Proteomics Core analyzed:

- 60 frozen tissue normal
- 60 frozen tissue tumor
- 61 tumor plasma samples with 81 unpaired normal samples. Normal samples were provided by IRB-approved study 00001316.

Stage III and IV patients were excluded. Due to the limited number of samples in this pilot cohort, we trained models in an LOO fashion for every analyte separately. During the train phase, we performed feature selection, missing data imputation and normalization; the same transformations were then applied to the validation sample (the LOO sample) using the means and variance learned on the train data. For certain analytes, we performed preliminary, analyte-specific transformations and feature selection. We utilized binary end points at the time of our analysis, 21 October 2021: DS, deceased at the time of analysis.

### Clinical data analysis

We collected 74 plasma and tissue samples of patients with clinical stage Ia, Ib, IIa and IIb, resectable pancreatic adenocarcinoma. We obtained clinical characteristics and longitudinal clinical and surgical pathology information for each patient whose sample was analyzed for our multi-omic analysis (Supplementary Table 1). Our baseline model for the clinical and surgical pathology analytes included general features such as self-reported sex, age, body mass index/weight/height, tumor stage/size, histological grade, pathological variable (margin status, grade, pathological staging, perineural invasion and lymphovascular invasion), treatment duration and type, family history and personal history of comorbid conditions, including other cancers.

### NGS targeted genomics

Bulk tissue samples were processed via NGS Tempus xT oncogene panel, specifically a v4 xT assay covering 648 genes, spanning ~3.6 Mb of genomic space at 500× coverage. An industry-standard bioinformatics pipeline was run on the NGS data for alignment, quality control and calling of somatic SNVs, INDELs and CNVs. SNVs were counted per gene in the target panel, generated via Freebayes snp calling pipeline with matched tumor–normals, resulting in 611 gene-level SNV features. INDELs were counted per gene in the target panel, with INDEL calling via the Pindel pipeline using matched tumor–normals, resulting in 126 gene-level INDEL features. Additionally, called CNVs were counted per gene in the target panel, resulting in 648 CNV features. Upon obtaining gene-level somatic SNVs, INDELs and CNV features, further feature preprocessing was performed, specifically univariate normalization, pruning of low-variance features (with variance threshold <0.05) and dropout of highly correlated features (Spearman correlation coefficient <0.95). Processed genomic features consisted of 337 somatic SNV, 219 CNV and 72 INDEL gene-level features, respectively considered for predictive patient survival outcome models.

### RNA sequencing

Whole-transcriptome sequencing (RNA-seq) was performed on 72 tumor tissue samples. In addition, we used 204 (out of 382 total) RNA-seq pancreatic tissues samples from the GTex consortium as controls. The GTex samples were selected using the following criteria: participant did not have a cancer diagnosis and participant’s age was matched to the age range of the pilot cohort. We then derived two types of RNA-seq features:

1. Gene-level estimated read counts for a set of genes that we found to be differentially expressed between cancer and noncancer samples.
2. Read counts per gene for a set of fusion genes.

We obtained estimated transcript read counts by running Kallisto tool (v.0.46.1) on the fastq files for cancer and noncancer samples. We aggregated transcript-level read counts to gene-level counts using tximport R package (v.1.14.2, Bioconductor v.3.10); this step reduced the number of features from 169,000 transcripts to 30,427 genes.

To further reduce the feature space and retain only the most promising features, we ran a differential expression analysis between cancer and noncancer samples. First, we removed all counts below 2 and then removed any genes (separately for cancer and noncancer datasets) for which fewer than 25% of samples in the set had nonzero values. This left us with 16,470 genes for the cancer set and 10,478 genes for the noncancer set. We then only kept genes in the intersection of noncancer and cancer gene sets, leaving us with 10,185 genes in total. We selected 2,000 genes with the lowest adjusted *P* values using the default analysis in DESeq2 package (v.1.26.0). Finally, we trained our classifiers using log_10_ estimated read counts for these 2,000 genes as features. Unpaired differential expression was conducted via Mann–Whitney *U*-test with *P* value < 0.05, from which the 2,000 most differentially expressed RNA gene transcripts were selected.

Fusion gene derivation from RNA-seq data was another category of omic features considered in the study to capture translocations, interstitial deletions or chromosomal inversions of two distant, independent genes. Fusion gene features were derived from RNA-seq data using an alignment-free algorithm^52^. The number of reads mapping to each fusion gene was aggregated, then limited to known COSMIC fusion pairs. In total 29 fusion gene features were derived from tumor tissue RNA-seq data.

### Proteomics and lipid analysis

Proteomics analyses were performed on 58 patients with paired tumor–normal tissue samples, via resection of tumor and normal samples from the same frozen tissue block and on 61 tumor plasma samples with 81 unpaired normal samples (Supplementary Table 16). Proteomics data were generated using data-independent acquisition mass spectrometry (DIA-MS) technology, with post-processing bioinformatics pipelines performing quality control, peak picking, retention time alignment, scoring and false discovery rate identification, normalization and quantitation^53^. MS2 peak areas at both protein and peptide levels were computed as proteomics features, using a 3,777-protein panel for paired tumor–normal tissue samples and a 1,052-protein panel for unpaired plasma samples. Similarly, lipidomics analysis using the Lipidyzer Platform kit with internal lipid class standards for quantification reference was performed on plasma samples to obtain composition and concentrations for lipid species, lipid classes and fatty acids.

Further pre-processing steps for all proteomics and lipidomics data included filtering out proteins and lipids with more than 25% missing data not meeting quality control criteria, removing proteins with a low variance <0.1 threshold, followed by imputation of remaining missing values using halved median values for each column and univariate normalization of each column. Alternative strategies for imputation of missing proteomics values, specifically column mean and *k*-nearest neighbor imputation, were deemed too sensitive to outliers due to the small sample size.

Differential expression analysis was performed on the 58 paired tumor–normal tissue samples. A Wilcoxon rank-sum test was performed between the dependent tumor–normal proteomics samples, with a two-tailed *P* value < 0.05 threshold applied to further remove tumor tissue protein distributions similar to their respective paired normals.

Differential expression analysis was performed on the 61 tumor plasma samples with unpaired 81 plasma samples. A Mann–Whitney *U*-test was performed between unpaired tumor–normal protein distributions, with a two-tailed *P* value < 0.05 threshold applied to remove plasma tumor protein distributions similar to the unpaired normals.

Overall, proteomics and lipidomics analysis generated 3,777 tumor tissue proteomic, 1,051 plasma proteomic and 939 lipidomic features. Plasma proteomic features were reduced to 257 via tumor–normal plasma protein differential expression analysis (Mann–Whitney *U*-test, *P* value < 0.05). Redundancy was reduced by elimination of highly correlated features (Spearman correlation, rho < 0.95, *P* value < 0.05) leaving 406 lipidomic features. Tumor tissue proteomic features were pruned to 1,130 by eliminating those not expressed at higher levels in tumors compared to normal pancreas (Wilcoxon signed-rank test, *P* value < 0.05).

### Plasma proteomics methods

Aliquots from plasma samples were depleted of 14 highly abundant proteins using the High Select Top 14 Abundant Protein Depletion Camel Antibody Resin (ThermoFisher Scientific) according to the manufacturer’s protocol. Proteins from 5 μl depleted and undepleted plasma were then processed separately for tryptic digestion on the Protifi S-Trap columns according to the manufacturer’s protocol (Protifi). Mass spectrometry data were acquired on an Evosep HPLC system in line with an Orbitrap Exploris 480 (ThermoFisher) instrument, operating in data-independent acquisition (DIA) mode, separately for the depleted and undepleted plasma samples. Full LC–MS instrument settings are provided with raw data in the public repository.

Peptide identification and quantification was performed as described previously^54^ using the OpenSWATH workflow and searched against the Human Twin population plasma peptide assay library. Proteotypic peptides were aggregated into quantitative protein abundance estimates using mapDIA software^55^. To generate a single table of quantified plasma proteins from each sample, we appended only unique proteins (for example, not seen in undepleted) from depleted runs to the undepleted plasma protein table.

### Plasma lipidomics methods

#### Sample processing and lipid extraction

Lipids were extracted from plasma using the Bligh-Dyer method as previously described^56^. The extracted dry lipids were resuspended in 250 µl running buffer (10 mM ammonium acetate and 50:50 methanol:dichloromethane). Lipids were analyzed on a Sciex Lipidyzer Platform with a standardized workflow for the simultaneous analysis of 1,153 lipids representing 13 lipid class. Samples were loaded by direct infusion from a Shimadzu LC-30AD LC system equipped with a SIL-30AC auto sampler. Lipid concentrations were determined by the Lipidyzer software using the ratio of the endogenous lipid to internal standard. Data are reported for each individual lipid species as an aggregated value for lipid classes and as the relative composition compared to all other measured lipid classes.

### Tissue proteomics methods

#### Sample processing and lysis

Tumor and nontumor tissue sections were homogenized in 8 M urea with 5% SDS and 100 mM glycine. Following sonication and centrifugation (1,500*g*) to pellet debris, the protein concentration was determined using a Pierce BCA assay (ThermoFisher Scientific). A total of 30 μg from each sample were then processed and digested using the S-TRAP micro-elution tips (Protifi) according to the manufacturer’s protocol. Peptides were analyzed using a ThermoFisher Ultimate3000 HPLC system in line with a Fusion Lumos Orbitrap mass spectrometer operating in DIA mode. Full MS details are provided with raw files in the public repository. Data were analyzed using our established openSWATH-based^57^ workflow using the pan-human library^58^ as previously described^54,59^. The mapDIA software^55^ was used to perform protein-level quantification from proteotypic peptides.

### Computational H&E slide analysis

#### Slides and computational pipeline overview

The 71 cases in our MT-Pilot cohort had available FFPE tumors that we used to prepare H&E slides for computational analysis. After slide digitization (Aperio GT450 scanner with ×40 objective), the resulting WSIs (*n* = 71) were loaded up to the slide viewer (ImageScope, Leica Biosystems) for a pathologist to box-outline random regions of interest (ROIs) with cancer cells for the analysis. Our goal was to extract architectural features of cancer cell nuclei and assess their fitness and contribution as an analyte in single- and multi-omic ML-based DS prediction models. The ROIs marked (*n* = 2,908) and exported from WSIs were subsequently analyzed by two semantic neural network models. The first (DeepLabV3Plus) provided a mask of cancer cells and the second (StarDist) provided a mask of all nuclei in the ROI (Fig. 2).

DeepLabV3Plus was trained and tested for the tumor cell masking task using biobanked digital H&E and IHC slides with PDAC. StarDist, a model that predicts cell nucleus instance using star-convex polygons, was already available^60^. The intersection of the masks yielded by these two models was the mask of cancer cell nuclei that we overlaid onto the ROI images.

#### Training and evaluation of DeepLabV3Plus model

Data for the DeepLabV3Plus training were obtained from ten slides (biobanked PDAC tumors at Cedars-Sinai) that we sequentially stained with H&E, digitized, destained, restained with IHC (pan-cytokeratin and DAB) and digitized again^61,62^. By overlaying the H&E WSIs onto the corresponding IHC WSIs, we obtained the ground-truth of cancer cells in the H&E WSIs. Matching ROIs with tumor cells were extracted from the corresponding H&E and IHC WSIs and aligned by image registration^61,62^. The aligned ROIs (*n* = 416) were divided into non-overlapping 256 × 256-pixel tiles. To generate the ground-truth mask for cancer cells in the tiles, the DAB staining image was digitally deconvoluted and thresholded, and the resulting cancer cell mask was smoothened by mathematical morphology operators. The tiles were then augmented 15 times^63^ and a set of paired H&E and tumor cell mask tiles (*n* = 39,840) was prepared for DeepLabV3Plus training. Training hyperparameters such as the minibatch, initial learning rate, momentum and L2-regularization for stochastic gradient descent optimizer were set to 12 tiles, 0.005, 0.9 and 0.001, respectively. At the end of 75 training epochs, the overall accuracy of 97.5% was observed.

The trained DeepLabV3Plus was tested for the tumor cell detection ability on a PDAC tissue microarray (TMA) (PA483e, TissueArray). To prepare H&E and IHC WSIs from the TMA, the slides underwent the same staining/restraining/digitization protocol as for the training slides. The H&E and IHC TMA WSIs provided 80 H&E cancer mask ROI pairs to measure the accuracy, mIoU and F1-scores (tumor and nontumor) of the DeepLabV3Plus.

#### Nuclear features

Nuclear feature extraction was preceded by color deconvolution^64^ of the ROI to digitally separate the image of hematoxylin staining from eosin. Subsequently, the cancer cell nuclei mask was overlaid onto the hematoxylin image and features of morphology (size and shape) and hematoxylin staining texture were quantitated for each nucleus under the mask by means of the 63-feature library (Source Data Fig. 2 and Supplementary Table 7) that we assembled from available resources^65,66^. Nuclear features from tumor cell nuclei across all regions in the case were aggregated by means of order statistics: maximum, minimum, average, s.d. and 1st, 5th, 10th, 25th, 50th, 75th, 90th, 95th and 99th percentiles, thereby yielding 819 (13 × 63) unique features for each case. The *z*-scored case-level features were used to develop ML models for survival prediction. All features in the library were image rotation invariant.

For validation in the TCGA cohort, 33 diagnostic WSIs with PDAC (1 WSI per case) that closely corresponded to WSI specifications (×40 scanning magnification and compression quality of 70) of the MT-Pilot WSIs were downloaded. The TCGA WSIs were annotated for cancer ROIs (624 total, 20 ROIs per WSI) and tumor cell nuclei (137,617 total, 4,170 nuclei per WSI) automatically identified and delineated in the ROIs by our pipeline. Subsequently, features (*n* = 819) were extracted from tumor cell nuclei in the ROIs, *z*-scored and classified by the ML models predicting DS that we developed using features extracted from the MT-Pilot WSIs. Before feature extraction, the H&E staining coloration in the ROIs was digitally matched to that in the MT-Pilot WSIs.

### Statistics and reproducibility

There was no a priori power analysis for this study. We had 93 patient samples available, of which 74 patients had their samples pass quality control, where samples from patients were both; viable for comprehensive molecular testing across all analytes and patients had stage I and II resected PDAC. No statistical method was used to predetermine the study sample size. The experiments were not randomized. We applied LOO in-pilot cross-validation and independent dataset validations. The investigators who conducted the full multi-omic analysis were not blinded to allocation during experiments and outcome assessment; however, investigators who conducted molecular analysis of the feature sets for each analyte were blinded to the outcomes of patients. Data distributions were not formally tested; however, we have provided clinical data distributions (Supplementary Table 1).

### Development of machine-learning models

The goal of our study was to train an ensemble of ML classification models, ranging from simple linear models (SVMs) to more sophisticated random forests and neural networks. The ensemble of the predetermined models’ approach was used to assess the level of dependence of multi-omic features and the extent to which subtle, nonlinear, cross-feature dependencies would provide additional signal and predictive power for nonlinear models. Additionally, the model architecture and model hyperparameters were prespecified and fixed for the study due to the limited sample size in the study and sample size to feature imbalance. This was carried out to prevent overfitting and over-tuning of models on the study dataset, instead showing relative performance across classification techniques and demonstrating directional performance of each approach. The architecture and hyperparameters for each classification model, optimization technique and hyperparameters used in the study were implemented in the Python programming language listed in code availability. Depending on the validation scenario (internal MT-Pilot cohort or external cohorts), developed models were validated using either the LOO cross-validation (internal MT-Pilot cohort only) or using analyte combinations depending on their availability in the validation cohorts (TCGA, JHU cohort 1, JHU cohort 2 and MGH cohort).

### Validation cohorts

Four validation cohorts were utilized in the study. The TCGA, JHU cohort 1 and cohort 2 and MGH cohort. The TCGA and JHU cohort 1 are publicly available datasets^14,46^. JHU cohort 2 is an independent prospective cohort employing identical proteomic and lipidomic analysis as our MT-Pilot and whose raw data was analyzed utilizing the Molecular Twin ML model pipeline by the JHU team for validation.

### Reporting summary

Further information on research design is available in the Nature Portfolio Reporting Summary linked to this article.

### Supplementary information

Reporting Summary

Supplementary Tables 1–16 Table of contents and Supplementary Tables 1–16 in each tab.

### Source data

Source Data Fig. 1 Complete multi-omic dataset: statistical source data.

Source Data Fig. 2 Nuclear features and computational pathology source data for analysis.

Source Data Fig. 3 Multi-omic modeling results with feature weights and statistical source data.

Source Data Fig. 4 Feature correlations, top multi-omic features statistical source data, UMAP multi-omic feature weights and informative nodes.

Source Data Fig. 5 Parsimonious learning curve complete data and statistical source.

Source Data Extended Data Fig. 1 Multi-omic modeling results with feature weights and statistical source data.

Source Data Extended Data Fig. 3 Nuclear features source data.

Source Data Extended Data Fig. 4 TCGA full multi-omic analysis set.

## Data Availability

Transcriptomic, genomic and clinical data used in this study are available under National Center for Biotechnology Information/National Institutes of Health BioProject ID PRJNA889519 and the associated SRA database. Proteomic data used in this study were submitted and are available in the proteomics Identification Database (PRIDE) as ‘Profiling of pancreatic adenocarcinoma using artificial intelligence-based integration of multi-omic and computational pathology features’ under project accession no. PXD037038. Lipidomic data used in this study are submitted and are available in the MassIVE Dataset Repository project under accession no. MSV000093118. The human pancreatic adenocarcinoma genomic data were derived from the TCGA Research Network at http://cancergenome.nih.gov/. Previously published data from TCGA and JHU cohort 1 (refs. ^14,46^) that were re-analyzed here are available at 10.1038/s43018-023-00697-7 and serve as source data for Table 2. The complete multi-omic MT-Pilot dataset utilized for Table 1 is provided and is identical to the source data for Fig. 1. Source data are provided with this paper. All other data supporting the findings of this study are available from the corresponding author upon reasonable request.

## Code availability

Software resources utilized in this study are included in the reporting summary and associated code, as well as the architecture and hyperparameters for each classification model is available at 10.5281/zenodo.8423595.
