# Supplementary material for: The Molecular Twin artificial-intelligence platform integrates multi-omic data to predict outcomes for pancreatic adenocarcinoma patients
Source: Nat Cancer. 2024 Jan 22;5(2):299–314. doi: 10.1038/s43018-023-00697-7 (PMC10899109; doi:10.1038/s43018-023-00697-7)
Supplement: Supplementary file 1 — Reporting Summary [file 43018_2023_697_MOESM1_ESM.pdf]

# nature portfolio

Corresponding author(s): Dan Theodorescu

Last updated by author(s): Oct 14, 2023

# Reporting Summary

Nature Portfolio wishes to improve the reproducibility of the work that we publish. This form provides structure for consistency and transparency in reporting. For further information on Nature Portfolio policies, see our Editorial Policies and the Editorial Policy Checklist.

## Statistics

For all statistical analyses, confirm that the following items are present in the figure legend, table legend, main text, or Methods section.

| n/a | Confirmed | |
|---|---|---|
| ☐ | ☒ | The exact sample size (*n*) for each experimental group/condition, given as a discrete number and unit of measurement |
| ☐ | ☒ | A statement on whether measurements were taken from distinct samples or whether the same sample was measured repeatedly |
| ☐ | ☒ | The statistical test(s) used AND whether they are one- or two-sided<br>*Only common tests should be described solely by name; describe more complex techniques in the Methods section.* |
| ☐ | ☒ | A description of all covariates tested |
| ☐ | ☒ | A description of any assumptions or corrections, such as tests of normality and adjustment for multiple comparisons |
| ☐ | ☒ | A full description of the statistical parameters including central tendency (e.g. means) or other basic estimates (e.g. regression coefficient) AND variation (e.g. standard deviation) or associated estimates of uncertainty (e.g. confidence intervals) |
| ☐ | ☒ | For null hypothesis testing, the test statistic (e.g. *F*, *t*, *r*) with confidence intervals, effect sizes, degrees of freedom and *P* value noted<br>*Give P values as exact values whenever suitable.* |
| ☐ | ☒ | For Bayesian analysis, information on the choice of priors and Markov chain Monte Carlo settings |
| ☐ | ☒ | For hierarchical and complex designs, identification of the appropriate level for tests and full reporting of outcomes |
| ☐ | ☒ | Estimates of effect sizes (e.g. Cohen's *d*, Pearson's *r*), indicating how they were calculated |

*Our web collection on statistics for biologists contains articles on many of the points above.*

## Software and code

Policy information about availability of computer code

Data collection

Boto3 (v2.3.4) AWS Boto3 https://boto3.amazonaws.com/v1/documentation/api/latest/index.html
CSBDeep (v0.7.2) Weigert et al., 2018 http://csbdeep.bioimagecomputing.com/doc/
dplyr (v1.0.10) Wickham et al., 2022 https://github.com/tidyverse/dplyr
Freebayes (v1.3.6) Garrison et al., 2012 https://github.com/freebayes/freebayes
H5py (v1.13.2) The HDF Group https://github.com/h5py/h5py
Kallisto (v0.46.1) Brey et al., 2016 https://pachterlab.github.io/kallisto/about
Keras (v2.9.0) Tensorflow https://github.com/keras-team/keras
Matlab (v.2022b) N/A https://www.mathworks.com/products/matlab.html
Matplotlib (v3.5.2) Hunter et al., 2007 https://matplotlib.org/
NumPy (v1.21.0) Harris et al., 2020 https://github.com/numpy/numpy
Openslide (v1.2.0) Goode et al., 2013 https://openslide.org/api/python/
Pandas (v1.4.3) Pandas https://github.com/pandas-dev/pandas
Pathlib (v3.3) N/A https://docs.python.org/3/library/pathlib.html
PIL (v9.2.0) Clark et al., 2022 https://github.com/python-pillow/Pillow
Pindel (v0.2.5b9) Yi et al., 2009 http://gmt.genome.wustl.edu/packages/pindel/index.html
PORTs (v1.1, PORTS_20160411) Spraker et al., 2019 https://ncihub.org/groups/ports; https://ncihub.org/resources/downloads
PyEnsembl (v107) Howe et al., 2021 https://github.com/openvax/pyensembl
Python (v3.8.13) N/A https://www.python.org/
PyVCF (v0.6.8) N/A https://anaconda.org/bioconda/pyvcf
s3fs (v2021.7.2) Botocore https://pypi.org/project/s3fs/
Scikit-learn (v1.1) Pedregosa et al., 2011 https://github.com/scikit-learn/scikit-learn

SciPy (v1.9.0) Virtanen et al., 2020 https://github.com/scipy/scipy
StarDist (v0.8.3) Schmidt et al., 2018 https://pypi.org/project/stardist/
Tensorflow Data Validation (v1.9.0) Breck et al., 2019 https://github.com/tensorflow/data-validation
TxImport (v1.24.0) Soneson et al., 2015 https://bioconductor.org/packages/release/bioc/html/tximport.html
Varscan (v2.4.2) Koboldt et al., 2012 https://github.com/Jeltje/varscan2

Data analysis

Software resources utilized in this study are included above and associated code, as well as the architecture and hyper-parameters for each classification model is available at 10.5281/zenodo.8423595.

For manuscripts utilizing custom algorithms or software that are central to the research but not yet described in published literature, software must be made available to editors and reviewers. We strongly encourage code deposition in a community repository (e.g. GitHub). See the Nature Portfolio guidelines for submitting code & software for further information.

## Data

Policy information about availability of data

All manuscripts must include a data availability statement. This statement should provide the following information, where applicable:

- Accession codes, unique identifiers, or web links for publicly available datasets
- A description of any restrictions on data availability
- For clinical datasets or third party data, please ensure that the statement adheres to our policy

• Transcriptomic, genomic and clinical data used in this study is available in NCBI's/NIH BioProject: accession BioProject ID: PRJNA889519 and associated SRA database.
• Proteomic data used in this study was submitted and is available in proteomics Identification Database (PRIDE) as, Profiling of pancreatic adenocarcinoma using artificial intelligence-based integration of multi-omic and computational pathology features Project accession: PXD037038
• Lipidomic data used in this study is submitted and is available in the MassIVE Dataset Repository project accession: MSV000093118.
• The complete analytic MT-Pilot dataset for this study is also noted in Source Data.
• Validation of public data sets (TCGA and JHU Cohort 1) is available at DOI 10.5281/zenodo.10004026.
• Any additional information required to reanalyze the data reported in this work paper is available from the lead contact upon request.

## Human research participants

Policy information about studies involving human research participants and Sex and Gender in Research.

Reporting on sex and gender

Sex and Gender were based on self reporting. Our study incorporated nearly an even distribution of self reported sex, males and females. We had 47% females and 53% males. Findings are applicable to both sexes. Patient data is de-identified, but consent was obtained to share individual patient level data, including sex as a biologic variable, as noted in source data figure 1. In our all of our multi-omic analyses sex was also considered as a biologic variable.

Population characteristics

We collected 74 serum and tissue samples of patients with Stage 1a, 1b, 2a, and 2b, resectable pancreatic adenocarcinoma. Our cohort consisted of 64% patients with clinical Stage I disease and 36% patients with Stage II (Supplemental Table 1). We obtained clinical characteristics and longitudinal clinical information for each patient whose sample was analyzed for our multi-omic analysis. We collected information on sex, age, BMI/weight/height, surgical pathology features including: tumor stage/size, histologic grade, pathologic variables, as well as treatment duration and type, family history, and personal history of comorbid conditions including other cancers, all noted in Supplemental Table 2.

Recruitment

All patients were consented prior to specimen collection and all specimens were collected as part of standard of care and through protocol IRB STUDY00000806 MT-Pilot Study, Feasibility of Extensive Molecular Profiling of Pancreatic Tumors: Lessons for Molecular Twin. Patients were selected based on the samples that were available in the Cedars-Sinai Medical Center Biorepository. Tissues were procured from surgical specimens as part of the standard of care. Blood samples were collected with routine blood work. The time in which these samples were collected ranged from March 2015 to April 2019. Follow up data were completed based on the standard of care. All cases are pancreatic cancer with the diagnosis of ductal adenocarcinoma. This was chosen based on the availability of formalin fixed paraffin embedded (FFPE), frozen tissue, buffy coat, and plasma. FFPE and frozen tissue were collected following tumor resection and were stored in the biobank for future research use. The process of collection and storage was done on site at Cedars-Sinai Medical Center. We recognize this approach can lead to sample selection bias. However, we were able to find similar cohort of patients through our validation datasets to validate our study findings.

Ethics oversight

Cedars Sinai Medical Center Institutional Review Board. IRB STUDY00000806 MT-Pilot Study, Feasibility of Extensive Molecular Profiling of Pancreatic Tumors: Lessons for Molecular Twin.

Note that full information on the approval of the study protocol must also be provided in the manuscript.

# Field-specific reporting

Please select the one below that is the best fit for your research. If you are not sure, read the appropriate sections before making your selection.

☒ Life sciences ☐ Behavioural & social sciences ☐ Ecological, evolutionary & environmental sciences

# Life sciences study design

All studies must disclose on these points even when the disclosure is negative.

| | |
|---|---|
| Sample size | This was a pilot study, we included 74 patients with clinical Stage I (64%) and II (36%), surgically resectable PDAC |
| Data exclusions | We excluded stage 3 and 4 patients since this cohort had very few samples to train for prediction models. |
| Replication | Based on our findings of the multi-omic and parsimonious models in our pilot cohort we conducted a validation for DS prediction where we evaluated their predictive performance on an four independent testing datasets, the Cancer Genome Atlas (TCGA) containing 156 evaluable samples, JHU Cohort 1 containing 81 samples, JHU Cohort 2 containing 47 samples, and MGH Cohort containing 35 samples with similarly staged and treated PDAC. We successfully, externally validated our multi-omic panels with available and complete data accross four independent cohorts, TCGA, JHU Cohort 1, JHU Cohort 2 and MGH. |
| Randomization | There was no a priori power analysis for this study. We had 93 patient samples available of which 74 patients had their samples pass quality control, where samples from patients were both: viable for comprehensive molecular testing across all analytes and patients had stage I and II PDAC. The experiments were not randomized as we utilized all available samples. |
| Blinding | Blinding was not applicable to this study. The investigators who conducted the full multi-omic analysis were not blinded to allocation during experiments and outcome assessment. However, investigators who conducted molecular analysis for each of the feature sets of each analyte were blinding to the outcomes of patients. |

# Reporting for specific materials, systems and methods

We require information from authors about some types of materials, experimental systems and methods used in many studies. Here, indicate whether each material, system or method listed is relevant to your study. If you are not sure if a list item applies to your research, read the appropriate section before selecting a response.

## Materials & experimental systems

| n/a | Involved in the study | |
|---|---|---|
| ☒ | ☐ | Antibodies |
| ☒ | ☐ | Eukaryotic cell lines |
| ☒ | ☐ | Palaeontology and archaeology |
| ☒ | ☐ | Animals and other organisms |
| ☐ | ☒ | Clinical data |
| ☒ | ☐ | Dual use research of concern |

## Methods

| n/a | Involved in the study | |
|---|---|---|
| ☒ | ☐ | ChIP-seq |
| ☒ | ☐ | Flow cytometry |
| ☒ | ☐ | MRI-based neuroimaging |

## Clinical data

Policy information about clinical studies

All manuscripts should comply with the ICMJE guidelines for publication of clinical research and a completed CONSORT checklist must be included with all submissions.

| | |
|---|---|
| Clinical trial registration | NA |
| Study protocol | This was an internal protocol, IRB STUDY00000806 MT-Pilot Study, Feasibility of Extensive Molecular Profiling of Pancreatic Tumors: Lessons for Molecular Twin |
| Data collection | Our Molecular Twin Pilot Cohort (MT-Pilot) included 74 patients with clinical Stage I/II with surgically resected PDAC between March 2015 and April 2019. Clinical stage III and IV patients were not considered for inclusion. Tumor specimens were collected at the time of surgery and plasma specimens preoperatively. DS for all 74 patients within this cohort was recorded and treated as a binary endpoint at the time of our analysis, October 21, 2021. At this time, 45 (61%) patients were deceased. All demographic and clinical characteristics (Supplemental Table 1) were included as features for the clinical analyte in our multi-omic analysis. The surgical pathology information was obtained from the pancreas resection. Tumor and plasma specimens were assessed for individual features by molecular profiling including targeted next generation sequencing (NGS) DNA sequencing, full transcriptome RNA sequencing, paired (tumor and normal from same patient) tissue proteomics, unpaired (tumor from patients and normal unrelated controls) plasma proteomics, lipidomics, and computational.<br><br>Four validation Cohorts were utilized in the study. The Cancer Genome Atlas (TCGA), Johns Hopkins University (JHU) Cohort 1 and Cohort 2, and Massachusetts General Hospital (MGH) Cohort. TCGA and JHU are publicly available datasets. JHU Cohort 2 is an independent prospective cohort employing identical proteomic and lipidomic analysis as our MT-Pilot and whose raw data was analyzed utilizing the Molecular Twin MLA algorithm pipeline by the JHU team that we used for ML models validation. |
| Outcomes | Binary Endpoints: Disease survival (DS): deceased at time of analysis. |

Outcomes

For each specific model (ie Multi-omic, parsimonious) we determined rate of TP: True Positive, FP: False Positive, TN: True Negative, FN: False Negative, ACC: Accuracy, PPV: Positive Predictive Value, Sens: Sensitivity, Spec: Specificity.
